# Stat and interferon genes identified by network analysis differentially regulate primitive and definitive erythropoiesis

**DOI:** 10.1186/1752-0509-7-38

**Published:** 2013-05-15

**Authors:** Emily Greenfest-Allen, Jeffrey Malik, James Palis, Christian J Stoeckert

**Affiliations:** 1 Department of Genetics, Perelman School of Medicine, University of Pennsylvania, Philadelphia, PA, USA; 2 Center for Pediatric Biomedical Research, Department of Pathology and Laboratory Medicine, University of Rochester Medical Center, Rochester, NY, USA; 3 Center for Pediatric Biomedical Research, Department of Pediatrics, University of Rochester Medical Center, Rochester, NY, USA

**Keywords:** Primitive erythropoiesis, Definitive erythropoiesis, *Stat1*, *Stat3*, IFN-signaling, Gene-regulatory networks, Co-expression network inference

## Abstract

**Background:**

Hematopoietic ontogeny is characterized by overlapping waves of primitive, fetal definitive, and adult definitive erythroid lineages. Our aim is to identify differences in the transcriptional control of these distinct erythroid cell maturation pathways by inferring and analyzing gene-interaction networks from lineage-specific expression datasets. Inferred networks are strongly connected and do not fit a scale-free model, making it difficult to identify essential regulators using the hub-essentiality standard.

**Results:**

We employed a semi-supervised machine learning approach to integrate measures of network topology with expression data to score gene essentiality. The algorithm was trained and tested on the adult and fetal definitive erythroid lineages. When applied to the primitive erythroid lineage, 144 high scoring transcription factors were found to be differentially expressed between the primitive and adult definitive erythroid lineages, including all expressed STAT-family members. Differential responses of primitive and definitive erythroblasts to a *Stat3* inhibitor and IFNγ *in vitro* supported the results of the computational analysis. Further investigation of the original expression data revealed a striking signature of *Stat1*-related genes in the adult definitive erythroid network. Among the potential pathways known to utilize *Stat1*, interferon (IFN) signaling-related genes were expressed almost exclusively within the adult definitive erythroid network.

**Conclusions:**

*In vitro* results support the computational prediction that differential regulation and downstream effectors of STAT signaling are key factors that distinguish the transcriptional control of primitive and definitive erythroid cell maturation.

## Background

In adult mammals, red blood cells are ultimately derived from hematopoietic stem cells that commit to the erythroid lineage. Erythroid progenitors in the bone marrow give rise to a wave of morphologically identifiable precursors that undergo a limited number of cell divisions in association with macrophage cells. These maturing erythroblasts accumulate hemoglobin, reduce cell size, condense their nucleus and ultimately enucleate to form reticulocytes that are released into the bloodstream. Prior to birth, a similar process of “definitive” red cell production occurs in the fetal liver.

However, the embryo requires red blood cells prior to the formation of the liver. This need is satisfied by the emergence of a transient population of “primitive” erythroid cells from the yolk sac. In the mouse, primitive erythroid progenitors (EryP-CFC) first emerge in the yolk sac beginning at embryonic day 7.5 (E7.5), and generate a wave of maturing primitive erythroblasts that exclusively constitute red cells in the embryo until E12, when the fetal liver begins to release definitive erythrocytes [1]. Primitive erythroblasts progressively undergo nuclear condensation and accumulate increasing amounts of hemoglobin until replication ceases, ultimately reaching steady-state hemoglobin content and a final cell size more than six times that found in adult murine erythrocytes [2]. In the mouse, primitive erythroid precursors primarily express embryonic globins (βH1, ϵy, and ζ), while definitive erythroid cells in the fetal liver and bone marrow express adult globins (β1, β2, α1, and α2) [3,4]. Despite maturing in the bloodstream, primitive erythroblasts, like their definitive counterparts, ultimately enucleate to form reticulocytes [1].

Definitive erythropoiesis has been extensively studied and several key transcriptional regulators of erythroid cell maturation have been identified, particularly in the adult erythroid lineage produced in the bone marrow. However, relatively little is known about the regulation of primitive erythropoiesis. Some key transcription factors (TFs) have been identified that regulate the production of both primitive and definitive erythroid cells, including *Tal1*, *Lmo2*, *Gata1*, *Gata2*, and *Klf1*[5,6]. Other key TFs play lineage-specific roles; *c-Myb* and *Gfi1b*, for example, preferentially regulate definitive erythropoiesis [7-9]. Likewise, the targeted disruption of the cytokine erythropoietin (*Epo*) and its receptor (*Epor*) have revealed an essential role for this pathway in the synthesis of definitive erythrocytes. This is not the case for primitive erythropoiesis, where a reduced population of primitive erythroblasts continues to mature in the bloodstream of *Epo-* and *Epor*-deficient embryos [10-12].

Our goal is to utilize knowledge of definitive erythropoiesis to gain further insight into the mechanisms that regulate primitive erythroid maturation and to identify factors that may distinguish the maturation of these two distinct, but closely related erythroid lineages. We employ a network-based systems approach to infer lineage-specific transcriptional regulatory networks from annotated microarray expression data. These data were obtained from primitive erythroid, fetal definitive erythroid and adult definitive erythroid cells isolated from mouse embryos, fetuses, and adult bone marrow, respectively [13]. Five independent samples of primary erythroid precursors at three progressive stages of maturation (proerythroblasts, basophilic erythroblasts, and poly-orthochromatic erythroblasts), as well as reticulocytes, were purified by flow cytometry and used for the analysis of global gene expression on an Affymetrix platform.

Gene-interaction networks inferred from patterns of co-expression have become increasingly popular tools for exploring gene function in biological systems. Such analyses have largely focused on identifying functionally enriched integrated sub-networks of co-expressed genes representing coherent functional units or biological pathways [14-16]. However, the architecture of an interaction network also provides insight into specific gene essentiality in the modeled system. In particular, the topological prominence of a gene or protein in an interaction network may reflect its biological role [17-19], although the association between specific measures of topology (e.g., degree, centrality, or connectivity) and essentiality likely varies [20].

Here, we applied a three-stage semi-supervised machine learning algorithm to estimate gene essentiality during erythroid precursor maturation. We employed the well-characterized transcriptional control of definitive erythropoiesis to identify topological features of inferred transcriptional regulatory networks and patterns of gene expression during erythroid precursor maturation that characterize known key regulators of red cell differentiation. Using these features, we predicted potential regulators of primitive versus definitive erythropoiesis and these predictions were then validated experimentally. Taken together, our data indicate that differential STAT signaling plays an important role in the regulation of primitive compared to definitive erythropoiesis.

## Results

We identified 1,080 potential transcriptional regulators expressed in the microarray expression dataset of erythroid cells using Gene Ontology (GO) annotations (see Materials and Methods). Of this set of potential key factors, 16 were known to play either essential or non-essential roles in the regulation of adult definitive erythropoiesis and were used as a reference dataset for training the machine learning algorithm.

Lineage-specific regulatory networks were assembled by integrating factor co-expression and computational predictions of TF binding based on sequence similarity. Although less than 15% of the potential interactions were realized, the networks did not exhibit scale-free topologies. Networks were overall highly connected, with degree distributions left-skewed and most genes having >400 neighbors (Additional file 1: Figure S1). The full list of inferred interactions comprising these networks can be accessed through interactive search strategies on the ErythronDB website (http://www.cbil.upenn.edu/ErythronDB/search.jsp). No single pattern of expression or standard measure of topological prominence in the estimated regulatory networks characterized the reference gene set, although most were preferentially expressed in the more immature proerythroblast and basophilic erythroblast stages of maturation (Additional file 1: Figure S2).

We hypothesized that factor essentiality in highly-connected small-world networks might be better inferred by considering both expression data and multiple aspects of network architecture. To this end, we identified 11 properties that capture aspects of expression, differential expression and network topology (Table 1). Using a genetic algorithm (GA), we evolved a weighted sum of these properties that defined an essentiality score capable of segregating key TFs from no-impact or non-essential TFs.

**Table 1:** Measures of expression and network topology that potentially characterize the essentiality of a transcription factor in a gene-regulatory network

**Topological property**	**Abbrev.**	**Description**	**Reference**
Global Connectivity	GC	number of local networks in which gene is found	
Degree	*k*	number of edges incident to a gene, normalized by size of the neighborhood (*k*/N)	
Proportion Predicted Targets	T	proportion of incident edges annotated by predicted binding	
Weighted Clustering Coefficient*	CC_w_	$CC_{i}=\frac{E_{i}}{k_{i}\left(k_{i}-1\right)}$	[42]
Weighted Closeness Centrality^†^	C	$C_{i}=\frac{n-1}{\Sigma_{j\in V}{\setminus_{}}_{i}d_{G}\left(i,j\right)}$	[43]
Absolute Expression			
Proerythroblast	P	Average expression across replicates	
Basophilic Erythroblast	B	Average expression across replicates	
Polyorthochromatic Erythroblast	O	Average expression across replicates	
Reticulocyte	R	Average expression across replicates	
Differential Expression			
Profile Shape^‡^	PS	Best-fit vector mapped to the average expression profile	
Maximum Ratio	MR	Ratio of maximum to minimum expression during developmental series	

* ratio of the number of edges that exist between the *k* neighbors of gene *i* (*E*_*i*_) and the number of potential edges between the neighbors, or *k*_*i*_(*k*_*i*_ − 1).

^†^ reciprocal of the mean shortest path length between a gene *i* and all other genes that can be reached from it, where *V* is the set of all possible genes and *d*_*G*_ is the shortest path length between genes *i* and *j*. Path lengths were estimated using the Dijkstra distance [44] as implemented in the Boost Graph Library [45].

^‡^ Average expression profiles mapped to 28 templates using vector projection. The best-fit profile (PS) was used as an indicator of the trend in expression during erythropoiesis.

During most runs, the GA successfully converged on optimal solutions in less than 100 generations and performed well for both the training (adult definitive) and testing (fetal definitive) erythroid datasets. Various runs were differentiated by the GA parameters (e.g., mutation, cross-over rate, population size). Evolved weights comprising the best solutions found by the GA in each run were ranked by the product of their fitness in both the training and testing datasets and the top 10 solutions are listed in Additional file 2: Table S3. These results are representative of all solutions, which were highly consistent in highlighting measures of global centrality, clustering coefficient, out-degree, and average absolute expression in the basophilic and poly-orthochromatic erythroblast stages as important properties for discriminating key regulators.

**Figure 1:**
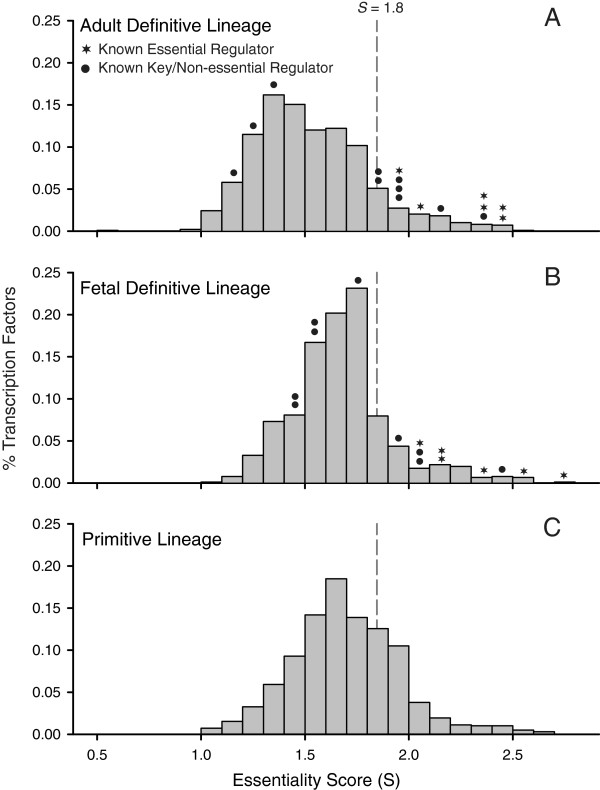
**Estimates of gene-essentiality inferred from expression and network topology discriminate key regulators of erythropoiesis.** Erythroid lineage-specific estimates of ranked gene-essentiality were made for each transcription factor expressed in the erythropoiesis expression dataset. The distribution of scores in all three erythropoietic lineages (**A**: adult definitive; **B**: fetal definitive; **C**: primitive) are strongly right skewed. Known definitive regulators (indicated by black dots and stars) exhibit a bi-modal distribution, with essential and key regulators, including *Klf1*, *Gata1* and *Tal1* (black stars), falling in the right tail and non-essential factors (e.g., *Nfia*) falling near the distribution mode. Thus, right-tail genes in all distributions (*S* ≥1.8) most likely possess topological and expression properties most similar to those of the known essential regulators of adult definitive erythropoiesis.

Using the weighted linear equation generated by the best solution (GA run 16–4; Additional file 2: Table S3) a lineage-specific essentiality score (*S*) was calculated for each TF. In training runs, the GA was unable to find a solution that grouped all known regulators, but instead consistently produced a solution in which there is a bi-modal split between two sets of TFs. All known essential regulators, including *Klf1*, *Gata1* and *Tal1*, cluster in the right tail of the strongly skewed score distribution; non-essential TFs fell closer to the modal value (Figure 1A). In the fetal definitive erythroid lineage, essential and non-essential factors were discriminated, but not as well differentiated as in the adult definite erythroid lineage (Figure 1B). Estimated essentiality scores for genes present in both adult definitive and primitive erythroid lineages are significantly correlated (*r*^*2*^ = 0.542, *p*-value < 0.001). The distribution of scores for all lineages were strongly right skewed and essential or key TFs known to play a role in all three erythroid lineages (e.g., *Klf1*, *Gata1*, *Tal1*, *Gata2*) consistently fell in the right tail (Figure 1). Thus, we hypothesized that right-tail genes (*S* ≥1.8) possess topological and expression properties most similar to those of the known essential regulators of adult definitive erythropoiesis and segregated them for further analysis. Erythroid lineage-specific essentiality scores are available in Additional file 3 (Tables S4-S6).

**Figure 2:**
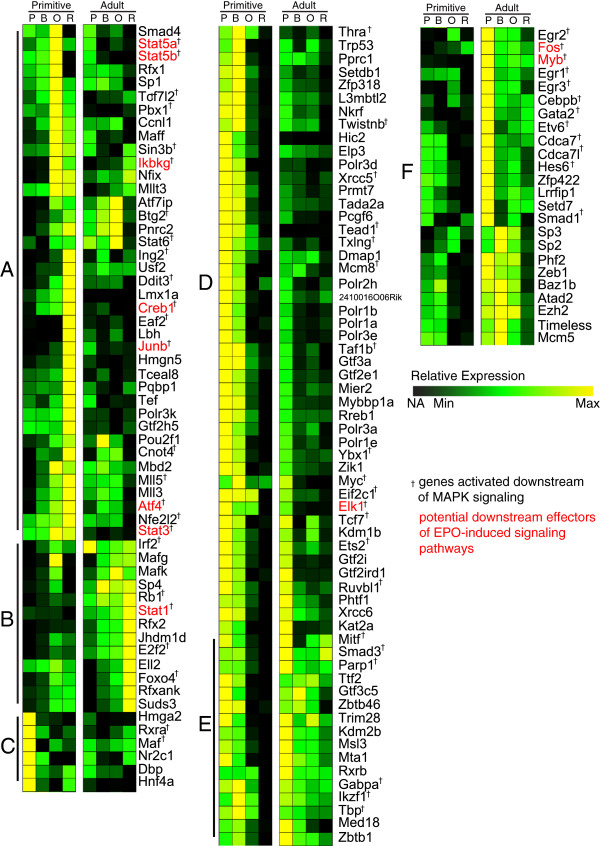
**Putative key regulators of primitive erythropoiesis are differential expressed in primitive versus adult definitive erythropoiesis.** 144 genes in the right-tail of the distribution of estimated essentiality scores for the primitive erythroid lineage were found to be differentially expressed compared to the adult definitive erythroid lineage. These genes fall into 6 clusters: **A**) primitive: preferentially expressed late, definitive: steady-state expression at low levels or not expressed; **B**) primitive: steady-state expression at low levels or not expressed, definitive: preferentially expressed late; **C**) preferentially expressed in primitive proerythroblasts; **D**) primitive: preferentially expressed early and not expressed late, definitive: expression at low levels with some preferential expression in proerythroblasts; **E**) primitive: preferentially expressed early and not expressed late, definitive: preferentially expressed early and expressed late; and **F**) primitive: preferential expression early at low levels or not expressed, definitive: preferential expression early or high-levels of steady-state expression. Gene set enrichment analysis identified 56 genes activated downstream of MAPK signaling (†), 11 of which also are downstream effectors of EPO-induced signaling pathways (red). A high-quality version of this image with the heatmap assembled into a single column and cluster dendrogram is available in the Additional file 4: Figure S7.

There are 252 transcription factors in the right-tail of the primitive erythroid score distribution, of which 144 were found to be differentially expressed, based on ranked cosine similarity, between the adult definitive and primitive erythroid expression datasets. Differentially expressed genes fall into six main groups, distinguished by the pattern of expression in early (proerythroblast to basophilic erythroblast) versus late (poly-orthochromatic erythroblast to reticulocyte) stages of erythroid maturation (Figure 2). A complete listing of these genes is available as an interactive search strategy from ErythronDB (http://www.cbil.upenn.edu/ErythronDB/im.do?s=aca0d8d30e3e95cb). Of the known key definitive erythroid regulators used to train the genetic algorithm, only *Gata2*, *Stat5a*, and *Stat5b* are differentially expressed between the two lineages.

Using the Database for Annotation, Visualization and Integrated Discovery (DAVID) v6.7 [21,22], we annotated and surveyed functional term enrichment in the differentially expressed result set. The 1,080 TFs used to build the interaction networks were employed as the background set for this analysis. Functional enrichment clustering using the DAVID resource resulted in a few significant groups, the most relevant of which included hemopoeisis and erythrocyte homeostasis, embryonic morphogenesis, regulation of cell cycle and cell differentiation, regulation of apoptosis, intracellular signaling, and a variety of signaling pathways (e.g., TPO, TGF-Beta, JAK-Stat, Wnt, ERBB, IL6 signaling). Closer examination of the DAVID results revealed a large degree of overlap in enrichment cluster membership and many of the TFs found in multiple enrichment clusters were known to be involved in or downstream effectors of MAPK signaling. Further scrutiny of the differentially expressed result-set revealed a total of 56 genes (~39%) related to MAPK signaling (Figure 2, indicated by †).

**Figure 3:**
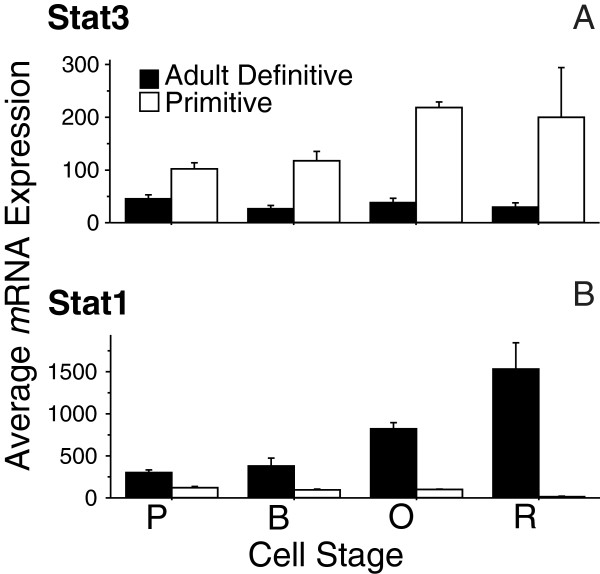
***Stat3 *and *Stat1 *exhibit erythroid lineage specific expression.***Stat3* (**A**) is expressed at low-levels only in the primitive erythroid lineage (white bars). In contrast, *Stat1* (**B**) is preferentially expressed in the adult definitive erythroid lineage (black bars), with expression levels increasing as cells mature.

Because EPO-induced MAPK signaling plays an important role in erythroid maturation, we looked for overlap between the MAPK-enriched gene set identified via the DAVID analysis and canonical EPO pathway genes using the Ingenuity Knowledge Base (Ingenuity® Systems, http://www.ingenuity.com). This list included both core pathway genes and those involved in downstream EPO-induced signaling pathways (e.g., PIK3/AKT, NFκB, and ERK/MAPK). We identified eleven TFs differentially expressed between primitive and adult definitive erythropoiesis that are potential downstream targets of EPO-signaling (Figure 2, highlighted in red). Interestingly, this list includes all but one of the STAT-family genes (*Stat1*, *Stat3*, *Stat5a*, and *Stat5b*) expressed in our erythroid lineage datasets. *Stat5a* and *Stat5b* were expressed during both primitive and definitive erythropoiesis, but exhibited increasing expression during the maturation of primitive erythroid cells and the opposite pattern during the maturation of adult definitive erythroid cells (Figure 2). *Stat3* was preferentially expressed in primitive erythroid cells and *Stat1* highly expressed only in the adult definitive erythroid lineage, with expression levels increasing as maturation proceeded (Figure 3). The remaining STAT-family gene expressed in our dataset, *Stat6*, was also identified by the GA as a potential regulator of primitive erythropoiesis and differentially expressed in the primitive compared to adult definitive erythroid lineage, but was not distinguished by the functional enrichment analysis.

**Figure 4:**
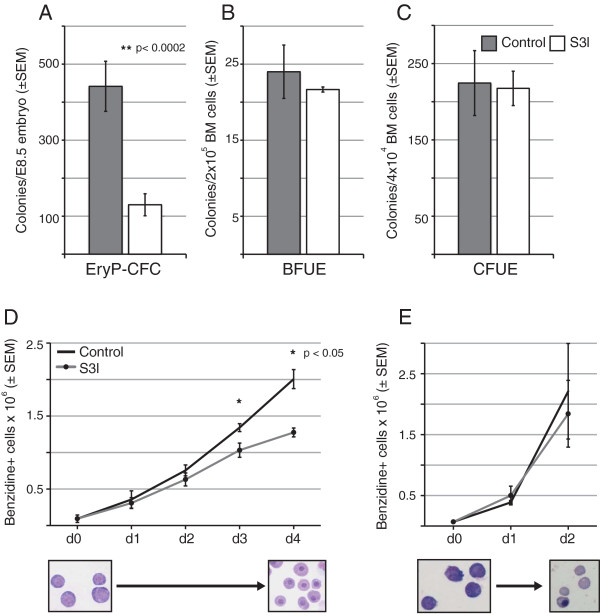
***Stat3 *inhibition differentially affects primitive erythroblast maturation *in vitro.*** Erythroid progenitor assays were performed on single-cell suspensions of individual, dissociated E8.5 embryos (**A**) and bone marrow (**B**,**C**) cultured in methylcellulose with appropriate media and cytokine supplementation. EryP-CFC (**A**) colonies were scored after 5 days, d3 BFU-E (**B**) were scored after 3 days and CFU-E (**C**) were scored after 2 – 3 days of culture. Primitive erythroblast maturation cultures were performed on cells pooled from dissociated E8.5 embryos (**D**) while definitive cultures were initiated with ESRE (**E**). All cultures were treated with DMSO as a vehicle control or 100 μM *Stat3* inhibitor, S3I-201. Liquid cultures (**D**,**E**) were pretreated with DMSO or S3I-201 for 2 hours prior to EPO stimulation. Definitive erythroblasts are cultured for 2 days, versus 4 days for primitive erythroblasts, because of their more rapid maturation *in vitro* and in vivo. Images are representative of primitive erythroblasts at days 1 and 4 of culture (**D**) and definitive erythroblasts at days 0 and 2 of culture (**E**).

Erythroblast maturation can be recapitulated *in vitro* using either liquid cultures or semisolid media that supports the generation of clonal erythroid colonies derived from erythroid progenitors. We took advantage of both liquid cultures and colony assay systems to test the function of *Stat3* in the primitive and definitive erythroid lineages using S3I-201, a small molecule inhibitor of *Stat3* dimerization [23,24]. Culture of primary yolk sac cells in the presence of the *Stat3* inhibitor S3I-201 reduced the number of EryP-CFC colonies by 70% (Figure 4A). In contrast, the formation of colonies from bone marrow-derived definitive erythroid progenitors, d3 BFU-E and CFU-E, was unaffected by *Stat3* inhibition (Figure 4B,C). Addition of the *Stat3* inhibitor also reduced the number of maturing primitive erythroblasts in liquid culture; definitive erythroblast production was not affected (Figure 4D,E). These data suggest a functional role for *Stat3* in primitive, but not definitive, erythropoiesis.

**Figure 5:**
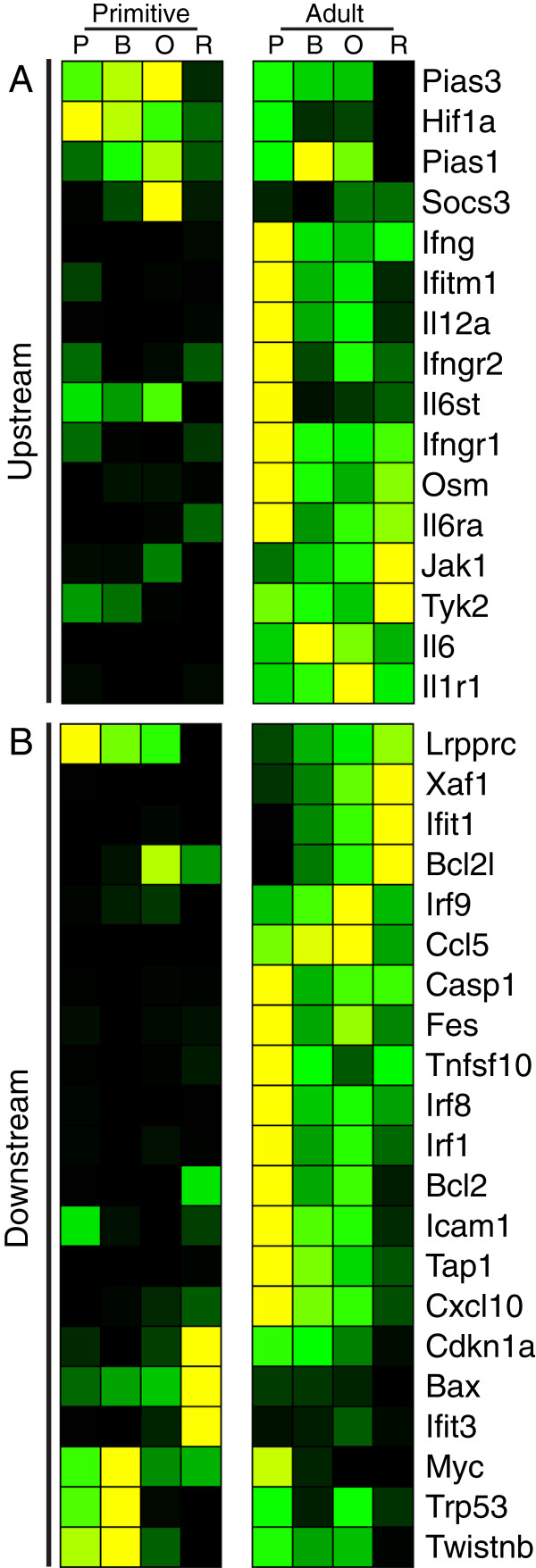
**The molecular signature for IFN signaling is specific to the adult definitive erythroid lineage.** In the microarray expression dataset, genes involved upstream (**A**) or downstream (**B**) of IFN-signaling were found to be preferentially expressed during adult definitive erythropoiesis. This includes *Ifng* and downstream apoptotic (*Casp1*, *Tnfsf10*) and anti-apoptotic genes (e.g., *Bcl2l*, *Bcl2*).

**Figure 6:**
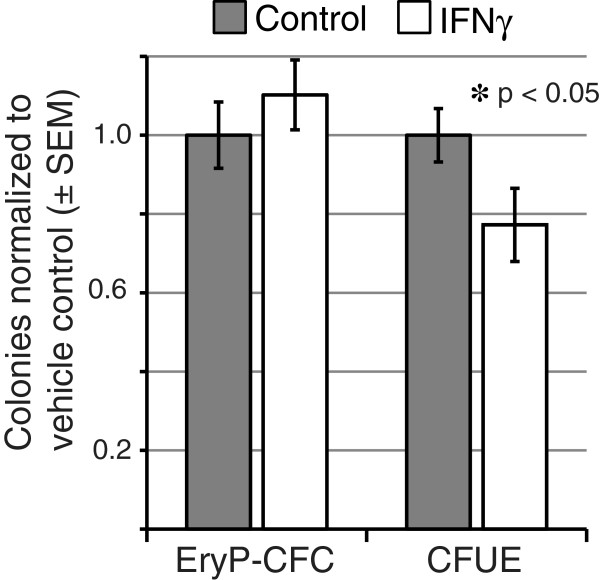
**IFNγ differentially modulates definitive erythroid colony formation.** Erythroid progenitor assays were performed on single-cell suspensions of dissociated murine E8.5 embryos (EryP-CFC) and adult bone marrow (CFU-E) in the presence or absence of IFNγ (10 ng/ml). CFU-E and EryP-CFC colonies were identified by morphologic criteria and scored at 2–3 and 5 days, respectively. Values for IFN-treated cultures are presented as normalized to vehicle-treated (phosphate buffer) control cultures.

We examined our erythroid lineage-specific datasets for upstream activators known to utilize *Stat1* as a mediator of signaling. A significant molecular signature of interferon signaling was found exclusively in the adult definitive erythroid lineage (Figure 5). Because IFNγ is known to inhibit colony formation of bone marrow-derived erythroid progenitors [25,26], we treated definitive and primitive erythroid colony-forming cultures with IFN As expected, IFNγ inhibited bone marrow-derived CFU-E colony formation by 20%. Consistent with the preferential expression of interferon genes in definitive erythroblasts, the addition of IFNγ to cultures of primary yolk sac cells did not affect the numbers of EryP-CFC-derived colonies (Figure 6). These expression and functional data indicate that interferon signaling regulates definitive, but not primitive, erythropoiesis.

## Discussion

The primitive, fetal definitive, and adult definitive erythroid-specific gene-interaction networks inferred from microarray expression datasets are highly connected and do not exhibit scale-free topologies. The connectivity of the inferred networks is, in part, a reflection of the underlying dataset, which is high quality and exhibits very high fidelity among replicates in the primitive and adult definitive erythroid lineages [13], effectively yielding stepped expression profiles with four stage-specific plateaus. The connectivity also reflects the underlying biology. By restricting our gene set to transcription factors, we segregated a single cohesive functional sub-network of the genome-wide expression during the terminal maturation of each lineage; i.e., the transcriptional regulation of erythropoiesis.

Annotating network edges with predicted TF binding potentials reduced the connectivity of the co-expression network by introducing directionality. However, the utility of this annotation was limited by the availability of partial weight matrices and binding consensus sequences, which only allowed predictions of targets for a third of the TFs considered in this analysis. These out-directed edges were important for discriminating essential from non-essential regulators (*W*_*T*_ ≥ 0.88 in all GA runs, Additional file 2: Table S3), suggesting that integrating further directionality would highlight additional differences among these lineages. The predicted binding may have introduced a bias to the analysis; genes for which binding targets were predicted were more likely to be identified as potential regulators, but only if many of their potential targets were present in the networks. For example, targets were predicted for *Foxo3*, but <1% of those targets (n = 3) were found in the adult definitive erythropoiesis network (Additional file 1: Figure S2B). The gene still had a relatively high essentiality score within the adult-definitive lineage (*S* = 1.96), determined by the other properties contributing to the score estimate.

Another limiting factor to this analysis was the use of the Gene Ontology to identify potential regulators. Due to the incompleteness of the annotation, some known, and likely several unknown, factors that play a key role regulating erythropoiesis were removed from consideration. For example, *Lmo2*, a known transcription factor and essential regulator of erythropoiesis, was filtered from the analysis due to the incompleteness of its GO annotation at the time the analysis was performed.

Despite these limitations, this system provided a rare opportunity to compare a set of closely related regulatory networks underlying the development of phenotypically distinct but functionally equivalent cells within a single organism. The essential regulatory mechanism underlying the fetal and adult definitive erythroid lineages has been well characterized, but comparatively little is known about the regulation of primitive erythropoiesis. The regulatory networks underlying these three erythroid lineages are different. However, they must also possess some commonalities as each results in the synthesis of a cell containing a complex cytoskeletal network, filled with hemoglobin, and devoid of a nucleus and internal organelles. While the timing and identity of essential regulators may vary, it is likely that they regulate the same or a similar suite of down-stream targets. Thus, we hypothesized that the topological and expression properties that characterize the known regulators of definitive erythropoiesis also should characterize equivalent regulators of primitive erythropoiesis; i.e., prior-knowledge about the definitive erythroid lineages could be used to test and validate computational predictions and then to moderate novel inferences about the regulation of the primitive erythroid lineage. With this in mind, the problem of predicting essential regulators of primitive erythropoiesis was considered a good fit for machine learning approaches and a task-specific algorithm was developed.

Our results revealed that key transcription factors in the definitive erythroid lineages could be discriminated by a combination of traits encompassing both the raw expression pattern and the architecture of the computationally inferred gene-interaction network. As expected, given the lack of the scale-free topology in our networks, local degree (*k*) provided little predictive power for identifying key regulators (*W*_*k*_ = 0); however considering specific subsets of these connections (e.g., global connectivity, number of predicted targets) did assist in discriminating the reference gene set. Measures of cohesion (clustering coefficient; *W*_*CC*_ = 0.98) and shortest-path centrality (*W*_*C*_ = 0.14) were also informative for the highly inter-connected networks (Table 1).

Overall, the estimated essentiality score for a gene in the adult definitive erythroid lineage was not a good predictor of its score in the primitive erythroid lineage. Additionally, known essential and non-essential definitive erythroid regulators were not as well-differentiated in the fetal dataset as in the adult, emphasizing that the majority of genes were not consistently ranked between the lineages. This is not surprising as a subset of these reference regulators (e.g., *Klf1*, *Gfi1b*) are known to play different roles in the primitive versus definitive erythroid lineages; thus the scores of individual genes are expected to vary across the lineages and likely reflect the underlying biology. This observation was supported by our analysis; ~57% of the predicted potential key transcriptional regulators of primitive erythropoiesis are differentially expressed in primitive compared to adult definitive erythropoiesis.

The list of putative key transcriptional regulators of primitive erythropoiesis predicted by the GA and found to be differentially expressed between primitive and adult definitive erythropoiesis was enriched in genes activated downstream of MAPK-signaling. This included a striking signature of genes in the EPO signaling pathway, including the STAT-family genes. It has been shown in cell culture that EPO activates *Stat1*, *Stat3*, and *Stat5a/b*[27]. *Jak2*-mediated phosphorylation of *Stat5a/b* is a core pathway mediating the EPO effect in erythroid cells; Jak2-deficiency in mice recapitulates the *Epo-* and *Epor*-null phenotype with an absolute block in definitive erythroblast production and fetal death by E12.5 [28]. STAT5-deficient fetuses ultimately develop severe anemia and die in the perinatal period [29], but show no absolute block in definitive erythropoiesis or any known primitive erythroid defect, suggesting that other transcriptional regulators are also involved in mediating this critical signal and supporting our computational prediction of a differential role for STAT-signaling in primitive compared to definitive erythropoiesis.

*Stat1* exhibits a pattern of increasing expression during erythroblast maturation specifically in the adult definitive erythroid lineage. Consistent with our computational finding, adult *Stat1*-null mice exhibit reduced numbers of CFU-E and elevated erythroblast apoptosis [26]. There is no known effect of *Stat1* deletion on primitive erythroblasts. Furthermore, *Stat1* has been implicated as a necessary downstream mediator of IFNγ in the negative regulation of bone marrow erythropoiesis [26] and IFNs α, β, and γ have all been shown to negatively regulate definitive erythropoiesis [25,30-32]. We find that genes involved in interferon-signaling are preferentially expressed in the adult definitive erythroid lineage (Figure 5), including *Ifng*, downstream apoptotic (*Casp1*, *Tnfsf10*) and anti-apoptotic (e.g., *Bcl2l*, *Bcl2*) genes, and genes involved in the negative regulation of cell proliferation (e.g., *Cxcl10*). This differential expression signature finds functional validation in our *in vitro* studies, which revealed that IFNγ inhibits definitive, but not primitive, erythroblast maturation.

The presence of *Stat3* in our list of putative regulators was especially interesting as it is expressed at extremely low levels in the microarray dataset (Figure 3) and was, in fact, filtered out of prior analyses due to its low expression level [13]. This gene was likely identified by the genetic algorithm as a putative regulator based on its topological prominence in the inferred regulatory network, emphasizing the information gained from considering multiple aspects of network topology. *Stat3* is preferentially expressed in primitive erythropoiesis, with expression levels increasing gradually during later maturation stages (Figure 3). Although it has been shown that EPO induces tyrosine phosphorylation of *Stat3*[33,34] and a potential role for this gene has been inferred in fetal definitive erythropoiesis through pathway analysis [35], activation of *Stat3* is uncommon in hematopoietic cell lines [27]. Here, the computationally predicted functional role for *Stat3* in primitive, but not definitive, erythroid cell maturation is validated *in vitro.* Small molecule inhibition of *Stat3* dimerization resulted in reduced numbers of erythroblasts late in the primitive erythroid culture, consistent with the increased expression of *Stat3* during late stages of primitive erythroblast maturation.

## Conclusions

Although primitive and definitive erythropoiesis share fundamental transcriptional regulators and result in the synthesis of terminally mature enucleated erythrocytes [1], they are fundamentally different processes. Definitive erythropoiesis in the adult is in steady state, continuously undergoing fine-tuned positive and negative regulation to maintain normal oxygen-carrying capacity. In contrast, primitive erythropoiesis emerges from the yolk sac and must transiently produce exponentially increasing numbers of erythroblasts to fill the newly-formed embryonic vasculature. We have identified the differential usage of *Stat1* and *Stat3*, as well as interferon signaling, as defining characteristics of these lineages that may reflect opposing roles in the regulation of erythroid cell proliferation and survival.

## Methods

### Microarray datasets

The expression data used in this analysis were obtained from Affymetrix Mouse430_2 chip mRNA expression data from four progressive stages of erythroid maturation, specifically the proerythroblast (P), basophilic erythroblast (B), polychromatic/orthochromatic erythroblast (O), and reticulocyte (R) stages from three erythroid lineages: primitive, fetal definitive, and adult definitive (Array Express E-MTAB-1035) [13]. Five biological replicates were performed for each maturational cell stage. Expression data were gcRMA normalized and MAS5 calls used to flag probe sets as expressed in the dataset only when present in a minimum of 3 out of 5 replicates for at least one maturational stage. Probe sets assigned an absent (A) call and any whose expression did not vary across replicates were also removed. Probe sets were mapped to EntrezGene identifiers and gene-level expression determined as the average across related probe sets.

### Predicted transcription factor binding

Potential binding sites were predicted for 352 TFs by matching partial weight matrices (PWMs) to sequences within 1 kb up- or downstream of the promoter regions of all genes expressed in the microarray data. PWMs were obtained from the public version of TRANSFAC^©^ and the freely available JASPAR databases. In addition, the CCNCNCCCN consensus sequence was used to identify potential targets of *Klf1*, a known key regulator of erythropoiesis [36]. Motif and consensus sequence matching was performed using the Transcription Element Search System (TESS) [37]. A maximum likelihood that a predicted site is a true binding site, or stringency, threshold ≥0.70 was adopted to identify the most likely predicted binding interactions between TFs and potential targets. The stringency of the best scoring match between a motif and matched sequence was used as a measure of binding potential (*b*_*ij*_) between the transcription factor (*i*) and predicted target (*j*).

### Network construction

Within each lineage, Pearson correlation (*r*) was used as a measure of co-expression between the ordered expression profiles of all expressed gene-pairs across the set of 20 samples (5 replicates per cell stage). The significance of correlations was assessed using a *t*-statistic and those supported by a *p*-value ≤ 0.05 were used to estimate an interaction network by drawing edges between all significantly correlated gene-pairs. Self-associations and weak correlations (|*r*| < 0.50) were dropped. Edges were assigned a base-weight of |*r*_*ij*_|, or the absolute value of the Pearson correlation between factors *i* and *j* and then weighted by the estimated binding potential, *b*_*ij*_, between the two genes. Interactions supported solely by co-expression were treated as undirected.

Expression data, profiles, predicted transcription factor binding, and the inferred regulatory networks used in this analysis are all accessible through ErythronDB (http://www.cbil.upenn.edu/ErythronDB), a fully searchable public resource on murine erythrocyte maturation.

### Machine learning identification of key regulators

Of genes expressed in the microarray dataset, we identified 1080 as putative transcriptional regulators using the Gene Ontology by selecting genes annotated by the following GO identifiers: GO:0003700, GO:0006350 and GO:0006351. We further identified eleven (11) properties (see Table 1 for a full listing), encapsulating aspects of expression, differential expression, and network topology that provide some insight into both the role and relative importance, or essentiality, of these transcription factors in the study system. Topological properties used in this analysis were chosen to capture multiple aspects of network architecture including local cohesiveness (clustering coefficient), shortest-path lengths (closeness centrality), and global dominance (degree centrality). In addition to these properties, we also considered other measures of dominance (e.g., betweenness centrality, or the number of shortest-paths on which a gene falls), and cohesiveness (e.g., the transitivity ratio, or the relative number of closed triplets associated with a gene), that were more computationally intensive. However, these measures did not well discriminate essential and non-essential regulators in initial trials and so not considered for the final analysis.

Lineage-specific values of each property were calculated for all TFs in expressed in our dataset. Values were then standardized to range from 0 to 1 to account for differences in scaling across the various measures.

It was not computationally feasible to assess the global topological prominence of each transcription factor in the estimated gene-interaction networks. Instead, fully-connected sub-networks for each TF and its neighbors were extracted and the topological properties for all TFs present in these “local-networks” calculated. We hypothesized that a key transcriptional regulator will be central and highly connected to its local network. We further postulated that essential factors should be prominent in the local networks of other key regulators as they likely serve as hubs between the connected sub-networks. Thus, here we take the modal value for each topological measure over all local-networks as an approximate measure of the global essentiality of the TF.

### Network topology

An essentiality score (S) was estimated as the weighted linear combination of these properties for each gene as follows:

$$S_{i}=\sum_{x\in X}W_{x}X_{i}$$

where *X* is the set of characteristics properties, and *x*_*i*_ is the value of property *x* for gene *i*. Property-specific weights, *w*_*x*_, were determined by using an unsupervised genetic algorithm. Genetic algorithms are commonly used search heuristics for parameter optimization and well suited to solve problems with a large search space [38]. The GA evolved populations of potential solutions, representing an individual solution as the numeric vector *W*, or the set of property-specific weights *w*_*x*_. Individual fitness was assessed using a non-parametric Kolmogorov-Smirnov test to evaluate whether the weighted score distinguished a reference set of 16 known definitive erythroid-associated transcriptional regulators. For the purpose of discussion, this TF reference set is split into three groups:

1. *Essential Regulators*: factors whose removal results in a complete block on hematopoiesis or erythropoiesis: *Tal1*, *Gata1*, *Myb*. *Lmo2* was not included as it was not annotated with the selected GO terms at the time of running the algorithm.

2. *Key Regulators*: factors whose removal produces severe defects or prevents terminal differentiation of definitive erythroblasts: *Klf1*, *Gfi1b*, *Zfpm1*, *Zbtb7a*, *Gata2*.

3. *Non-essential Regulators*: factors that affect stress erythropoiesis or related developmental processes (e.g., megakaryopoiesis) but do not block or otherwise disrupt terminal differentiation of definitive erythroblasts: *Foxo3*, *Ep300*, *Nfe2*, *Crebbp*, *Stat5a*, *Stat5b*, *Nfia*, *Fli1*.

The GA was trained on the dataset of expression values and local-network topology derived from the adult definitive erythroid microarray expression dataset. Best solutions were then tested by applying the weighted equation to the properties and network derived from the fetal definitive expression dataset. GA parameters were systematically adjusted (e.g., recombination and mutation rates, population size) and training repeated until the solutions were able to discriminate known regulators in both the training and testing datasets. The weighted ranking equation was then applied to the primitive erythroid dataset to predict novel regulators of that lineage.

### Hierarchical clustering

Lineage-specific log2 normalized expression profiles were clustered based on pairwise Pearson correlations. Hierarchical clustering and heatmap visualization were generated using GenePattern [39].

### Cross-lineage differential expression

The pairwise cosine similarity was calculated between the adult definitive and primitive erythroid expression profiles of each transcription factor. Similarity values were ranked and genes whose cosine similarity was less than or equal to the median value of the distribution (*cos* ≤ 0.92) were considered significantly differentially expressed during the maturation of adult definitive compared to primitive erythroid cells.

### Erythroid colony-forming assays

Outbred Swiss Webster mice (Taconic, Germantown, NY) were mated overnight and vaginal plugs checked the following morning (E0.3). E8.5 mouse embryos were dissociated with 0.25% trypsin (Worthington Biochemical, Lakewood, NJ, USA) to single cell suspensions and 1/10 yolk sac-equivalents were plated in duplicate in 1 ml IMDM, 1% methylcellulose (Stem Cell Technologies, Vancouver, BC, Canada), 5% PFHM-II (Invitrogen, Carlsbad, CA, USA), 10% serum replacement (Invitrogen), recombinant human erythropoietin (EPO; Amgen, Thousand Oaks, CA, USA) (2 U/ml), SCF (120 ng/ml), 2 mM MTG (Sigma-Aldrich, St. Louis, MO, USA), 2 mM glutamax (Invitrogen). EryP-CFC-derived colonies were counted after 5 days of culture at 37°C and 5% CO_2_, as described previously [40]. Murine bone marrow was cultured at a density of 4–5 × 10^4^ cells/ml in 1 ml of IMDM, 1% methylcellulose, 5% PFHM-II, 10% plasma-derived serum (PDS; Animal Technologies, Tyler, TX, USA), 20% BIT (Stem Cell Technologies), EPO (0.3 U/ml), 55 μM 2-ME (Gibco), 2 mM glutamax at 37°C and 5% CO_2_. CFU-E-derived colonies were enumerated at day 2 or 3 of culture.

### Erythroblast maturation culture

Dissociated E8.5 embryos were cultured on 0.1% gelatin-coated plastic for 24 hours in primitive erythroid maturation media containing IMDM, 10% serum replacement, 10% PFHM-II, 2 mM glutamax, 150 uM MTG, 1% PDS, and 1 U/ml EPO. After 24 hours, the non-adherent, primitive erythroid cells were transferred to uncoated wells with fresh maturation media and cultured for up to a total of 4 days. Definitive, extensively self-renewing erythroblasts (ESRE) were generated as previously described [41]. ESRE were induced to terminally mature in IMDM, 10% PFHM-II, 5% PDS, EPO (2 U/ml), 150 uM MTG, 2 mM glutamax at 37°C and 5% CO_2_.

## Abbreviations

GA: Genetic Algorithm; GO: Gene Ontology; PWM: Partial weight matrix; TF: Transcription Factor.

## Competing interests

The author(s) declare that they have no competing interests.

## Authors’ contributions

EG-A and CS conceived and designed the computational approach. EG-A implemented and performed the computational and statistical analyses. JM conceived, designed, and performed the *in vitro* analyses. All authors helped to draft the manuscript. All authors read and approved of the final manuscript.

## Supplementary Material

Additional file 1: Figures S1 and S2 Illustrating the degree distribution of the inferred transcriptional regulatory networks and the topological and expression properties of the reference gene set, respectively. Click here for file

Additional file 2: Table S3 Enumeration of the top 10 solutions generated by the genetic algorithm for discriminating known essential regulators in the adult definitive and fetal definitive erythroid lineages. Click here for file

Additional file 3: Tables S4, S5, and S6 Which provide ranked lists of genes ordered by their inferred essentiality scores in the primitive, fetal definitive, and adult definitive erythroid lineages, respectively. Click here for file

Additional file 4: Figure S7 A high-resolution version of Figure 2, with the expression-heatmap assembled into a single column to enhance readability. A dendrogram supporting assignment of genes into clusters is also diagrammed. Click here for file
