# Supplementary material for: Stat and interferon genes identified by network analysis differentially regulate primitive and definitive erythropoiesis
Source: BMC Syst Biol. 2013 May 15;7:38. doi: 10.1186/1752-0509-7-38 (PMC3668222; doi:10.1186/1752-0509-7-38)
Supplement: Additional file 1: Figures S1 and S2 — Illustrating the degree distribution of the inferred transcriptional regulatory networks and the topological and expression properties of the reference gene set, respectively. [file 1752-0509-7-38-S1.pdf]

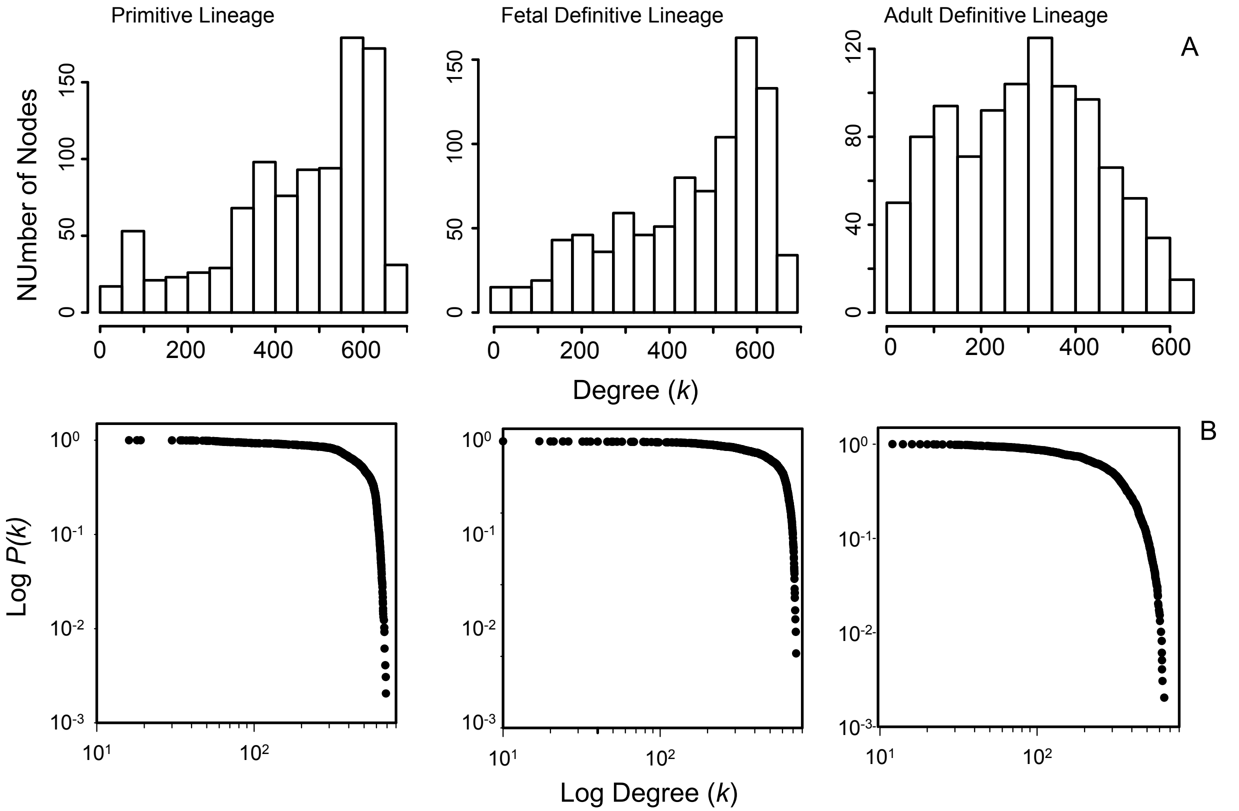

Figure S1. **Inferred erythroid lineage-specific transcriptional regulatory networks are not-scale free**.
A. Degree distributions for each network, emphasizing that most nodes have many connections.
B. The degree distributions do not follow a power law relationship; the proportion of nodes with a degree *k* [P(*k*)] is uncorrelated with degree (*k*).

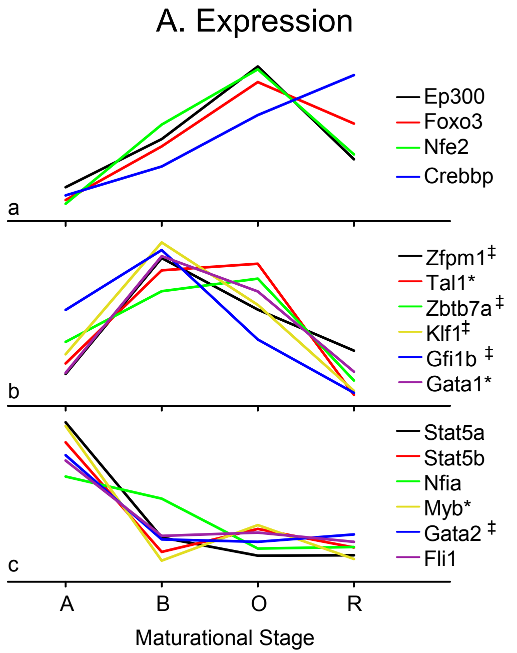

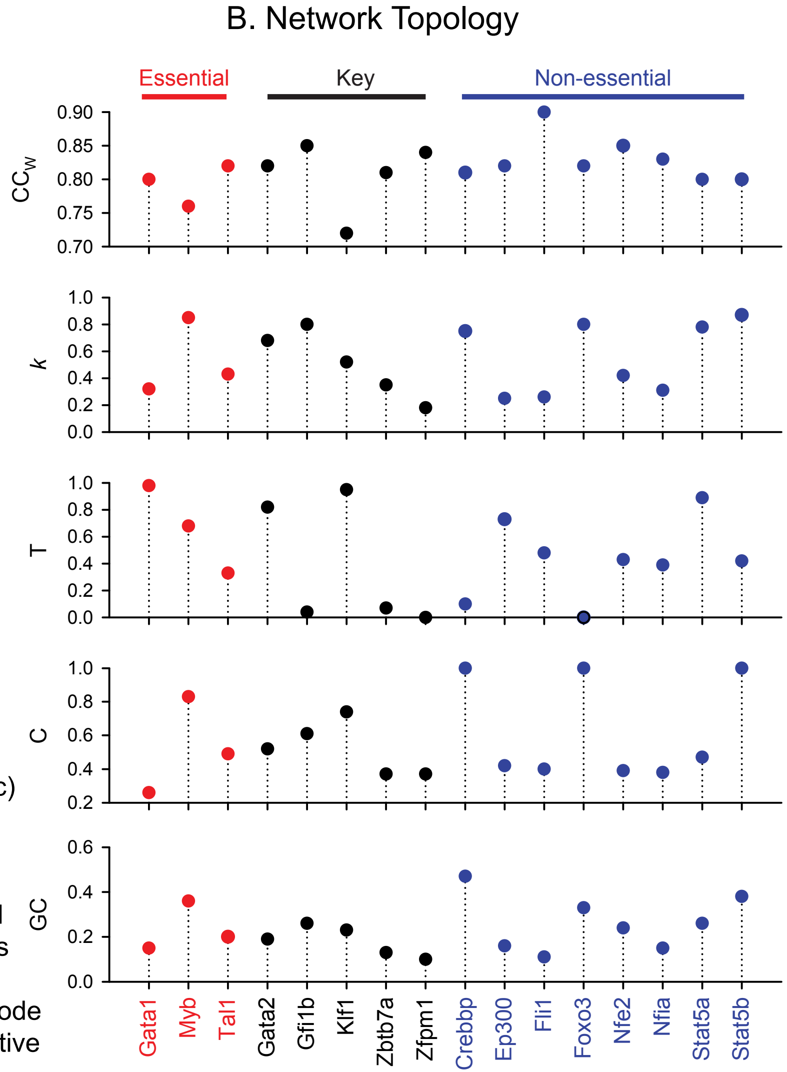

Figure S2. **No single property (expression or topological) well characterizes known transcriptional regulators of adult definitive erythropoiesis.**

A. Expression profiles: known regulators could be segregated into three classes (a,b,c) based on expression profile shape. Essential (*) and key (‡) regulators tend to be preferentially expressed during the initial stages of development.

B. A local neighborhood was extracted for each transcription factor (TF) in the inferred gene-regulatory networks. For each gene in a TF neighborhood, topological properties (clustering coefficient [CC_W], degree [k], out-degree [T], closeness centrality [C], global connectivity [GC]) were calculated. Values were normalized and, for each gene the mode of each set of property values across all TF neighborhoods was taken as a representative measure of topological prominence. No single value of any topological property well-characterizes the known regulators of definitive erythropoiesis, although clustering coefficients tend to be high (CC_W) and global connectivities (number of local neighborhoods in which a gene was present [GC]) tend to be low. Essential (red), key (black), and non-essential (blue) regulators also could not be distinguished by individual topological properties.
