# Supplementary material for: Stat and interferon genes identified by network analysis differentially regulate primitive and definitive erythropoiesis
Source: BMC Syst Biol. 2013 May 15;7:38. doi: 10.1186/1752-0509-7-38 (PMC3668222; doi:10.1186/1752-0509-7-38)
Supplement: Additional file 2: Table S3 — Enumeration of the top 10 solutions generated by the genetic algorithm for discriminating known essential regulators in the adult definitive and fetal definitive erythroid lineages. [file 1752-0509-7-38-S2.pdf]

Table S3. **Top-ten solutions generated by the genetic algorithm for discriminating known essential regulators**. For each run, weights were evolved for the 11 gene properties (expression: P, B, O, R; differential expression: PS, MR; and topological prominence: GC, CC_w, *k*, T, C) considered in this analysis (see Table 1). For each gene, normalized values of the traits were weighted and summed to calculate an essentiality score. Fitness of individual solutions was evaluated by how well each segregated known regulators of erythropoiesis using a Kolmogorov-Smirnov (KS) test. The equation was applied to both the adult and fetal definitive erythropoiesis datasets and ranked by minimizing the product of the test statistic in both datasets (Overall Fitness).

Run	GC	CC_w	*k*	T	C	P	B	O	R	PS	MR	Adult KS Statistic	Fetal KS Statistic	Overall Fitness
16-4	0.82	0.98	0	0.99	0.14	0.12	0.48	0.43	0.1	0.48	0.12	6.2E-07	2.8E-04	1.73E-10
32-4	0.75	0.69	0.02	0.96	0.15	0.06	0.54	0.59	0.02	0.51	0.02	6.8E-07	2.6E-04	1.76E-10
32-3	0.75	0.85	0	0.96	0.15	0.06	0.51	0.38	0.14	0.51	0.02	6.8E-07	2.8E-04	1.89E-10
16-1	0.82	0.62	0	0.99	0.14	0.08	0.48	0.43	0.02	0.48	0.03	6.2E-07	3.2E-04	1.99E-10
1-0	0.69	0.87	0.02	0.88	0.13	0.12	0.3	0.43	0.28	0.49	0.07	6.5E-07	3.1E-04	2.01E-10
1-3	0.69	0.69	0.02	0.88	0.12	0.05	0.29	0.43	0.28	0.49	0.05	6.5E-07	3.1E-04	2.01E-10
1-2	0.69	0.63	0.02	0.88	0.12	0.07	0.29	0.43	0.28	0.49	0.02	6.5E-07	3.2E-04	2.08E-10
1-1	0.69	0.74	0.02	0.88	0.12	0.03	0.29	0.43	0.28	0.49	0.04	6.5E-07	3.3E-04	2.15E-10
1-4	0.69	0.64	0.02	0.88	0.12	0.02	0.3	0.43	0.28	0.49	0.04	6.5E-07	3.3E-04	2.15E-10
16-0	0.9	0.52	0.01	0.99	0.14	0.03	0.2	0.83	0.21	0.68	0.04	6.0E-07	5.0E-04	2.96E-10
