# Supplementary figures and images for: Stat and interferon genes identified by network analysis differentially regulate primitive and definitive erythropoiesis
Source: BMC Syst Biol. 2013 May 15;7:38. doi: 10.1186/1752-0509-7-38 (PMC3668222; doi:10.1186/1752-0509-7-38)

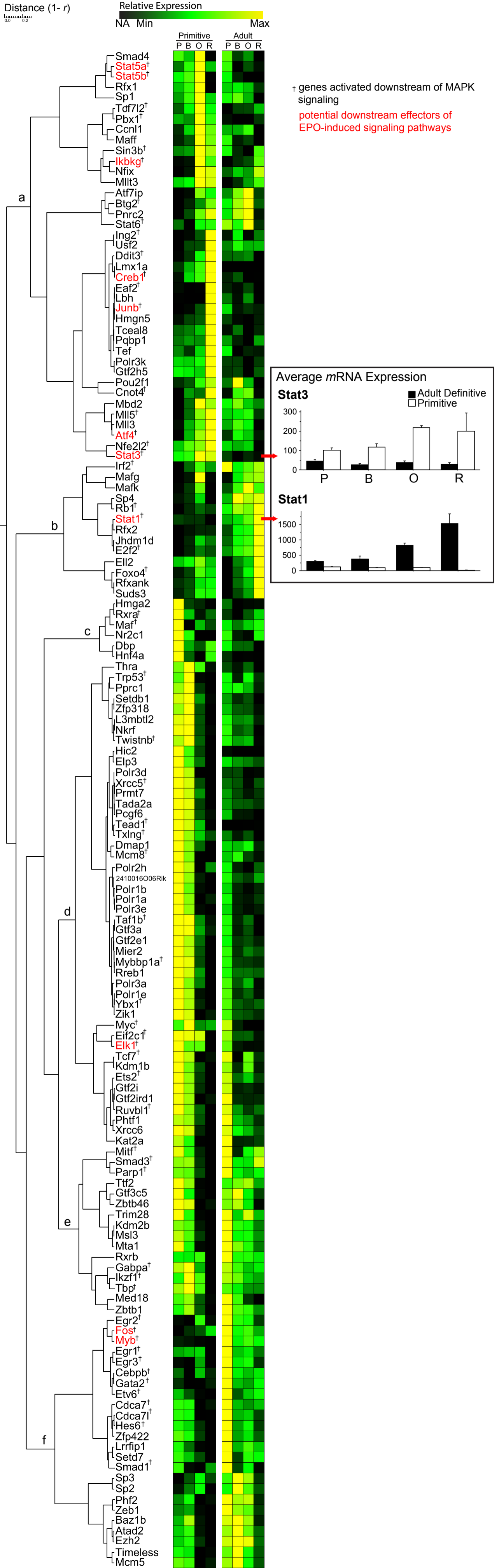

Supplement: Additional file 4: Figure S7 — A high-resolution version of Figure 2, with the expression-heatmap assembled into a single column to enhance readability. A dendrogram supporting assignment of genes into clusters is also diagrammed. [file 1752-0509-7-38-S4.pdf]
